# Supplementary material for: Feasibility and acceptability of gamified cycling exercise for residents in a long-term care home: A qualitative study
Source: PLoS One. 2025 Nov 6;20(11):e0335679. doi: 10.1371/journal.pone.0335679 (PMC12591412; doi:10.1371/journal.pone.0335679)
Supplement: Attachment — Submitted filename: Response to Reviewers.docx [file pone.0335679.s004.docx]

**Journal Requirements:**

1. When submitting your revision, we need you to address these additional requirements.

Please ensure that your manuscript meets PLOS ONE's style requirements, including those for file naming. The PLOS ONE style templates can be found at

<https://journals.plos.org/plosone/s/file?id=wjVg/PLOSOne_formatting_sample_main_body.pdf> and

<https://journals.plos.org/plosone/s/file?id=ba62/PLOSOne_formatting_sample_title_authors_affiliations.pdf>

**Feedback: Thanks for your suggestions. We make sure that our manuscript meets PLOS ONE’s style requirements.**

2. Thank you for stating in your Funding Statement:

This work was supported by the Canada Research Chair in Senior Care awarded to LH (Grant Number: GR021222). The funders had no role in the study design, data collection and analysis, decision to publish, or preparation of the manuscript. (<https://www.chairs-chaires.gc.ca/chairholders-titulaires/profile-eng.aspx?profileId=5178>)

Please provide an amended statement that declares *all* the funding or sources of support (whether external or internal to your organization) received during this study, as detailed online in our guide for authors at <http://journals.plos.org/plosone/s/submit-now>. Please also include the statement “There was no additional external funding received for this study.” in your updated Funding Statement.

Please include your amended Funding Statement within your cover letter. We will change the online submission form on your behalf.

**Feedback: Thanks for your suggestions. We have amended funding statement as follows and add “There was no additional external funding received for this study”:**

**Funding statement**

This work was supported by the Canada Research Chair in Senior Care awarded to LH (Grant Number: GR021222). The funders had no role in the study design, data collection and analysis, decision to publish, or preparation of the manuscript. (<Http://www.chairs-chaires.gc.ca/chairholders-titulaires/profile-eng.aspx?profiledld=5178>). There was no additional external funding received for this study.

3. Please amend either the abstract on the online submission form (via Edit Submission) or the abstract in the manuscript so that they are identical.

**Feedback: Thanks for your comments. We have reviewed both the abstract in the manuscript and the one in the online submission form and conform that they are identical. There are no amendments necessary.**

4. We note that Figure 1 includes an image of a participant in the study.

As per the PLOS ONE policy (<http://journals.plos.org/plosone/s/submission-guidelines#loc-human-subjects-research>) on papers that include identifying, or potentially identifying, information, the individual(s) or parent(s)/guardian(s) must be informed of the terms of the PLOS open-access (CC-BY) license and provide specific permission for publication of these details under the terms of this license. Please download the Consent Form for Publication in a PLOS Journal (<http://journals.plos.org/plosone/s/file?id=8ce6/plos-consent-form-english.pdf>). The signed consent form should not be submitted with the manuscript, but should be securely filed in the individual's case notes. Please amend the methods section and ethics statement of the manuscript to explicitly state that the patient/participant has provided consent for publication: “The individual in this manuscript has given written informed consent (as outlined in PLOS consent form) to publish these case details”.

If you are unable to obtain consent from the subject of the photograph, you will need to remove the figure and any other textual identifying information or case descriptions for this individual.

**Feedback: We securely filed the signed consent form in the individual’s case notes and revised the manuscript accordingly (page 12, line 186-187).**

5. Please review your reference list to ensure that it is complete and correct. If you have cited papers that have been retracted, please include the rationale for doing so in the manuscript text, or remove these references and replace them with relevant current references. Any changes to the reference list should be mentioned in the rebuttal letter that accompanies your revised manuscript. If you need to cite a retracted article, indicate the article’s retracted status in the References list and also include a citation and full reference for the retraction notice.

**Feedback: We review our reference list and ensure that it is complete and correct.**

**5. Review Comments to the Author**

**Reviewer #1:** The authors discussed the manuscript in a clear manner and in detail. Ample references were used to support their findings as well as in the literature review section. The research done was crucial to understand how exergames could benefit older community.

**Feedback: Thanks for your positive comments. Your comments are highly encouraging for us to explore the implementation of gamified exercise further.**

**Reviewer #2:** I have no competing interests.
The work was done perfectly. Also it is have few minor notes to adding: like statistical program and other statistical ways used in showing research results.
Secondly, the study should be built-in wider and large studying group.

**Feedback: Thanks for your comments. We report participant numbers by age range, gender distribution, ethnicity, dementia status, and staff roles using descriptive statistics (numbers and percentages). This information has been added to the Methods section (Page 11, Lines 170-172). Beyond that, no additional data is available for further statistical analyses.**

**This study represents the first phase of implementing gamified exercise in long-term care homes. We assessed its acceptability and feasibility in the current study and plan to explore its effectiveness in future research while expanding participation across diverse resident populations. We have addressed these limitations in Page 33, Lines 552-560, and outlined our future plans in the Implications (page 34, line 574) and Conclusion sections (page 36, line 604-605.**
