# Supplementary material for: Feasibility and acceptability of gamified cycling exercise for residents in a long-term care home: A qualitative study
Source: PLoS One. 2025 Nov 6;20(11):e0335679. doi: 10.1371/journal.pone.0335679 (PMC12591412; doi:10.1371/journal.pone.0335679)
Supplement: Attachment — Submitted filename: Response_to_Reviewers_auresp_2.docx [file pone.0335679.s005.docx]

Reviewers' comments:

6. Review Comments to the Author

Please use the space provided to explain your answers to the questions above. You may also include additional comments for the author, including concerns about dual publication, research ethics, or publication ethics. (Please upload your review as an attachment if it exceeds 20,000 characters)

Reviewer #3: The authors went through the comments from both reviewers and made the necessary changes in the manuscript. Also considered future application on a wider scale.

Response: Thanks for your comments.

Reviewer #4: Introduction
-Please include the definitions and specific context of feasibility and acceptability in your introduction. Acceptance of the motivated cycling exercise by the LTC home, patient, family member, LTC staff, or all the above? Feasibility of the motivated cycling experience to be implemented at a larger scale, or in a specific context, and how is feasibility being determined?

Response: Thanks for your comments. We have now defined acceptability as the extent to which residents, family members, and staff perceive the cycling game intervention as agreeable, engaging, and valuable. Feasibility is defined as the extent to which the intervention can be practically delivered within the long-term care settings, considering staffing, scheduling, and infrastructure. The study explores acceptability and feasibility from the perspectives of all key stakeholders—residents, family members, LTC staff and leadership. This comprehensive approach reflects the collaborative nature of care in LTC environments, where residents’ uptake and staff facilitation are closely intertwined. In terms of scope, our assessment of feasibility focuses on the implementation of the cycling game within the specific context of the participating LTC homes. We examine factors such as workflow integration, staff capacity, and resident suitability. While findings may inform future scale-up, this pilot study is primarily concerned with identifying facilitators and barriers within the current care environment. We added relevant content in Page 5 (lines 78-91).
-Unclear how the research questions directly relate to acceptance and feasibility of the cycling game.

Response: Thanks for your comments. We clarify that both research questions are designed to explore key dimensions of acceptability and feasibility (Page 5, lines 78-85). The first question examines older adults’ experience, which provides insight into acceptability from the resident perspective—whether they find the cycling game engaging, appropriate, and enjoyable. The second question captures the perspectives of family members, staff and leadership, offering critical insight into both the acceptability (e.g. perceived value) and feasibility (e.g., workflow integration, staff capacity, and operational fit) of implementing the intervention in LTC. These perspectives together inform the potential for sustained use and broader implementation.
Methods
-Please clarify which interview and focus group discussion questions address acceptance and which address feasibility. Factors of acceptance and feasibility can be mentioned to indicate if there is a focused approach.

Response: Thanks for your comments. We revised the manuscript to explicitly map these questions to the constructs of acceptability and feasibility (Page 8, lines 146-147, 154-156).
-Please confirm if the interviews were conducted with family members or patients or both. “After completing each exercise session (twice weekly for four weeks), we conducted 20-30- 143 minute individual conversational interviews with residents and their families.” Were multiple interviews conducted with each resident, one after each exercise session?

Response: Thanks for your comments. We confirm that interviews were conducted with both residents and family members; however, these were conducted separately due to differing schedules in this study setting. Each family member was interviewed individually at their preferred time points, while residents were interviewed after their final exercise session. The aim was to gather in-depth reflections on their overall experience with the intervention rather than session-specific feedback. We have clarified this in the revised manuscript to avoid confusion and to better reflect our data collection approach (Page 8, lines 159-163).
-Please clarify if the staff focus group discussions were mixed (multiple roles/ranks in one group)? Please also clarify if there was repetition in focus group discussion participation, such as one staff member attending multiple discussions.

Response: Thanks for your comments. We confirm that there was no repetition in focus group participation; each staff member attended only one discussion session. Focus groups were organized by role type for scheduling and comfort reasons. Specifically, allied health professionals participated in one group, managers in another, and nurses and care aides were grouped together in several sessions due to their different working sites. We have clarified this point in the revised manuscript (Page 9, lines 165-171).

-Did the researchers take into account the multiple levels of rank in one discussion group? Was the possibility of bias accounted for in the focus group discussions? For example a subordinate being uncomfortable to openly criticize the intervention in the presents of leadership which brought in the intervention.

Response: Thanks for raising this important point. We acknowledge the potential influence of hierarchical dynamics in focus group discussions. Our focus group structure also helps avoid this influence. Additionally, the topic of discussion, feasibility and acceptability, was not directly related to job performance or sensitive organizational matters. We have clarified this in the revised manuscript (Page 9, lines 165-171)).
Results
-Accessibility is the first the author is mentioning feasibility from the LTC home’s perspective, goes back to earlier question of clarifying the acceptance and feasibility parameters. Is the focus of the study to assess acceptability and feasibility by the patients or the care providers, or both? Staff discussing the interventions affect on the patient and how to bring the intervention to the patient is still patient focused, but I do not currently see how lines 257-260 provide evidence of feasibility/acceptability

Response: Thanks for your comments. The aim of our study was to explore acceptability and feasibility from the perspectives of all three groups: residents, family members, and care providers. The staff quote on lines 257-260 illustrates feasibility from the organizational and staffing perspective. Oliver’s comments specifically reflect considerations around leadership responsibility, structured implementation through the rehabilitation team, and the phased approach to scale the intervention across appropriate residents. While the content centers on patients, the insight being shared is fundamentally about how feasible it is for staff and the organization to deliver and expand the intervention.
-Which themes and sub themes speak to acceptability by the patient/LTC home versus which speak to feasibility for the patient/LTC? Why were the themes included? Please link the themes to research your questions relating to acceptability and feasibility.

Response: Thanks for your helpful comments. We have added a paragraph at the end of the results section to explicitly link the identified themes and subthemes to our research questions, as well as the concepts of acceptability and feasibility (Page 24, lines 459-477). This addition clarifies how each theme aligns with the experiences and perspectives of residents, families, staff and leadership, and enhances the overall coherence of the findings.
- Consider breaking up the results in feasibility and acceptability sections/subsections to ensure clear findings

Response: Thanks for your suggestion. While we recognize the value in distinguishing feasibility and acceptability in some research designs, we intentionally chose an integrated thematic approach in this study. Our rationale is that feasibility and acceptability are not mutually exclusive; rather, they are interconnected aspects that together inform the successful implementation of a gamified cycling program in LTC settings. For example, themes such as “Ease of use and Accessibility” speak directly to both the practicality of setup (feasibility) and user satisfaction (acceptability). Separating them could risk oversimplifying these nuanced intersections. We have clarified this rationale in the manuscript and ensured that each theme is linked to our research questions to highlight how they collectively reflect feasibility and acceptability (Page 24, Lines 459-477).
Discussion
-lines 513 mention as assessed through TAM, was the study using the TAM framework throughout? If so the TAM should be mentioned in the introduction, with its definitions and factors of acceptability

Response: Thanks for your comments. We did not use TAM framework to guide our thematic analysis; rather, our themes were developed inductively from the data. However, TAM is an important framework related to understanding acceptability, so we referenced it in the discussion section to help contextualize our findings and support interpretation.
-discussion highlights the perceived benefits of the cycling game thoroughly, but please also include detailed discussion of the other factors relating to acceptability and feasibility.

Response: Thanks for your comments. Beyond perceived health benefits, we also discussed ease of use as a key factor in acceptability and suggested design improvements to enhance user engagement and comfort (Page 27, lines 530-558). In terms of feasibility, we highlighted fun engagement and community building as important facilitators (Page 29, Lines 559-566), along with the role of families in addressing staffing challenges and supporting implementation (Page 29, lines 567-580). These factors collectively provide a more nuanced understanding of what supports or hinders the adoption and sustainability of gamified cycling in LTC settings.
Conclusion
-No comments

-The paper is surely contributing to implementation practices in elder care with innovate care technology. There is a clear contribution to knowledge in the areas of elderly care and the use of gamified technology in healthcare. The data was well collected, but the data is approached and presented without clear criteria on the definitions or factors of acceptability and feasibility.

Response: Thanks for your comments. We integrated these definitions and related factors into the manuscript. (Page 5, lines 78-91)
