# Supplementary material for: Feasibility and acceptability of gamified cycling exercise for residents in a long-term care home: A qualitative study
Source: PLoS One. 2025 Nov 6;20(11):e0335679. doi: 10.1371/journal.pone.0335679 (PMC12591412; doi:10.1371/journal.pone.0335679)
Supplement: Attachment — Submitted filename: Response_to_Reviewers_auresp_3.docx [file pone.0335679.s006.docx]

Reviewers' comments:

Reviewer #5: Hello author, thank you for providing this manuscript for review. I have shared several comments, primarily highlighting its strengths. However, I've also noted some areas where improvements could be made.

1. Technical Soundness and Data-to-Conclusion Alignment
The study is presented as a qualitative inquiry examining the acceptability and feasibility of a gamified cycling exercise program in a long-term care (LTC) setting. The authors carefully define essential concepts. For instance, in lines 78–91, "acceptability" is defined as how engaging and valuable the residents, family members, and staff find the intervention, whereas “feasibility" refers to practical aspects like staffing and scheduling. This dual focus is well justified in the context of the study.

The methodological approach is thoroughly described (e.g., an Interpretative Descriptive approach noted in lines 97–105 and the iterative coding process using NVivo discussed in lines 181–190). The methods for observations, individual interviews, and focus groups are suitably explained, and incorporating perspectives from multiple stakeholders (residents, families, staff, and leadership) enhances the conclusions drawn.

Quotations from participants (e.g., lines 245–254 and lines 378–391) effectively illustrate the emergent themes (such as ease of use, physical/mental health benefits, and enjoyment/community engagement) rooted in the data. Along with summary tables (Tables 1 and 2 referenced around lines 231 and 242), these data points provide credible support for the manuscript’s conclusions.

The conclusions regarding feasibility and acceptability (lines 638–650) are well grounded in the study findings and existing literature (e.g., lines 500–529).

Despite the overall strong technical design, it would be beneficial for the authors to include a brief discussion on sample size limitations (14 residents, 11 family members, and 34 staff) when generalizing findings, even though qualitative studies typically value depth over breadth. Moreover, incorporating a summary at the end of the Results section (around lines 459–477) that links each theme to the research questions would clarify how the data supports the conclusions on acceptability and feasibility.

Response: Thanks for your comments. We have added a brief reflection in the discussion addressing the sample size limitations (lines 622-627). We have also added a concise summary paragraph at the end of the results sections, explicitly linking each of the identified themes back to the research questions. We believe this addition improves the clarity and coherence of our findings (lines 468-494).

2. Appropriateness and Rigor of the Statistical (Descriptive) Analysis

The study mainly employs qualitative thematic analysis and uses descriptive statistics (frequencies and percentages, as seen in lines 191–198 and Table 1) to outline participant demographics. This approach is entirely suitable, considering that the study is not aimed at testing quantitative hypotheses.

Braun and Clarke’s six-step reflexive thematic analysis is well defined and seemingly followed rigorously. The iterative team coding with NVivo is clearly outlined (lines 181–190), reassuring the reader of the robustness of the thematic findings.

Descriptive statistics were employed to present demographics (lines 222–235), which is appropriate.

Given the qualitative nature of the study, advanced statistical analyses are not anticipated. However, the authors might briefly discuss how they ensured trustworthiness in the descriptive analysis (e.g., through triangulation, member checking, or inter-coder reliability). Such clarification would address potential concerns regarding methodological rigor. Integrating quantitative outcome measures (e.g., validated scales for mobility or affect), as suggested in the limitations section (lines 600–604), would also strengthen the study.

Response: Thanks for your comments. We have expanded the methods section to include a description of the strategies we used to ensure trustworthiness. (lines 190-192, 222-229)

3. Data Availability and Transparency
The detailed descriptions provided in the Methods (lines 157–174) and supplemental materials reveal that raw interview protocols, field notes, and coding schemas are accessible, which is commendable for a qualitative study. While the paper discusses data rigor (lines 213–220) and ethical protocols (lines 205–212), it does not explicitly state whether transcripts, coding frameworks, or anonymized data are available in a public repository.

Response: Thanks for your comments. We have added a clear statement to the ethical consideration section to clarify our approach. (lines 215-217). While full transcripts and anonymized datasets are not publicly available, we have included coding frameworks and selected de-identified excerpts in the manuscript to support transparency.

4. Clarity, Coherence, and Use of Standard English
The manuscript is well-structured and logically organized, featuring clear research questions (lines 92–96), a comprehensive methodology section, and results that flow logically.

The language throughout is accessible, standard, and grammatically correct, making it suitable for an international journal.

Complex concepts like feasibility and acceptability are well-defined and consistently applied (lines 78–91). The use of participant quotations enhances clarity and reader engagement (e.g., lines 243–249, 373–379). e.g., lines 243–249, 373–379).

Response: Thanks for your comments.

Reviewer #6: This manuscript addresses a highly relevant issue in the context of institutionalized elder care by exploring the feasibility and acceptability of a gamified exercise intervention. The qualitative approach is appropriate, and the study demonstrates strong implementation, particularly through the inclusion of multiple stakeholder perspectives: residents, family members, and care staff. The inclusion of participants with dementia and the thoughtful adaptation of the technological tool to their needs are important strengths. Overall, the study is meritorious and provides original evidence. Nevertheless, I suggest minor revisions aimed at clarifying and strengthening some theoretical and analytical elements, as outlined below.

Theoretical integration: use of multiple conceptual frameworks

Throughout the manuscript, several frameworks are referenced: the Consolidated Framework for Implementation Research (CFIR), the Technology Acceptance Model (TAM), and the Unified Theory of Acceptance and Use of Technology (UTAUT). However, the relationship among these frameworks is not entirely clear. The manuscript states that the analysis was guided by CFIR while also indicating that themes were developed inductively. In addition, TAM and UTAUT appear later in the discussion as contextual frameworks. This coexistence may generate confusion regarding the overarching epistemological orientation of the study. I recommend clarifying how these frameworks were integrated in the design, analysis, and interpretation stages.

Response: Thanks for your comments. Due to our focus on the implementation process of the gamified exercise intervention, we primarily used the CFIR to guide our thematic analysis. CFIR provided a structured lens to explore contextual, organizational, and individual factors influencing feasibility and acceptability within LTC settings. While other studies have employed TAM and UTAUT to examine technology adoption, these frameworks are not used in our data analysis. Instead, we referenced TAM and UTAUT in the discussion section as comparative models to assess whether our CIFR-informed themes aligned with constructs from these established technology acceptance frameworks. This comparison enabled us to interpret our findings within a broader body of literature, while maintaining an implementation-focused epistemological orientation. We made relevance revision to the discussion section (lines 547-582).

Distinction between acceptability and feasibility

While the authors argue that acceptability and feasibility are interconnected, the boundaries between the two are not always clearly defined in the manuscript. Specifically, the theme ease of use is repeatedly associated with both resident satisfaction (acceptability) and staff-related implementation concerns (feasibility). In such cases, it would be helpful to explicitly discuss how each concept is interpreted, the dimensions in which they manifest, and how they are empirically distinguished in the thematic analysis.

Response: Thanks for your comments. We have clarified how acceptability and feasibility are interpreted and distinguished in our thematic analysis. An explanation has been added to the end of the findings section to address this distinction (lines 486-491).

Generalizability and potential cultural bias in sampling

The sample appears to be demographically homogeneous, with all participants (residents, families, and staff) of Asian background, and the study was conducted in a single institution. Although the manuscript acknowledges limitations regarding generalizability, it does not explain why such a specific population was selected or what cultural or institutional implications this may have for interpreting the findings.

What was the rationale for selecting such a demographically homogeneous sample, particularly in terms of participants' cultural background? Was this decision deliberate, institutionally constrained, or a result of logistical limitations?

Response: Thanks for your comments. The demographic homogeneity of our sample was not an intentional selection but a natural consequence of recruiting at the available site. The LTC facility we partnered with serves a predominantly Chinese-Canadian community, which naturally shaped the participant composition. While this reflects the local institutional and cultural context, it was not a deliberate choice to limit diversity. We recognize that this homogeneity may influence the generalizability of our findings and have emphasized the importance of future research involving more culturally diverse populations and multiple sites to explore broader applicability and cultural nuances in acceptability and feasibility. We added this explanation to the limitation section (lines 619-622).
